# American Academy of Dental Science

**Published:** 1892-02

**Authors:** 

**Affiliations:** American Academy of Dental Science


### AMERICAN ACADEMY OF DENTAL SCIENCE.

THE American Academy of Dental Science held its regular monthly meeting in the Boston Medical Library Association rooms, October 7, 1891, President Seabury in the chair.

*President Seabury.*—" Inflamed Pulps requiring Extirpation; how best to treat?" is the first item on the card. Has any one any communication or paper on this subject?

*Dr. Banfield.*—The speedy removal of an inflamed pulp, giving the patient the minimum amount of discomfort, is a matter of considerable importance to ourselves as well as to our patients.

Its removal is accomplished by two general methods: First, through the action of some drug, thereby causing devitalization; second, through the use of probes and broaches. Few patients, however, are willing to submit to the latter barbarous operation. At the present time the destruction of the pulp by the first method is often attended with considerable disappointment, due probably to a stage of inflammation of the pulp which resists the action of the drug used.

A greater or less amount of pericementitis often ensues, rendering the use of arsenic, at this stage, of doubtful value. Our first thought, in cases of inflamed and aching pulps, is to apply some sedative, and, if possible, reduce the congestion. After such an application, we may still find the pulp in the course of a day or two very sensitive, and upon exposure to the air giving pain which is difficult to control. Unless very carefully inserted the application of arsenic at this stage is liable to cause pain, the pulp also resisting the action of the drug. It has been the custom of some operators, after an arsenical application has been removed and a remnant of pulp found to be still alive and sensitive, to use a combination of tannin and creosote, allowing it to remain for some weeks. I have, however, removed applications of this kind which have remained in nearly a year, and still found the pulp as sensitive as ever.

In view of the liability of extension of inflammation resulting in more or less pericementitis, it seems to me that whenever a pulp resists the action of arsenic or cocaine, the best treatment, for both patient and dentist, is to remove the pulp at once by probes, using an anæsthetic locally or otherwise, provided the pulp-canals are accessible.

We may hope that in the near future, through the use of cocaine or some combination of it with other drugs, we may have better success in treatment of and extirpation of the pulp.

*Dr. Williams.*—In the early days of my practice, Dr. Harwood always advocated what was called "butchering the pulp,"—that is, taking it out alive. That was before the days of anæsthetics, and Dr. Harwood was very successful in this heroic practice. This method was not attended with the irritability of the root which sometimes follows the application of arsenic.

When pulps are inflamed, the inflammation should first be reduced before an application is made to destroy its life. To reduce this inflammation, I have found nothing better than bicarbonate of soda applied to the cavity on a loose pledget of cotton, and the surrounding parts saturated with chloric ether. This application may be left in place from half an hour to a day according to the extent of the inflammation. After this treatment the pulp is less likely to give pain in applying arsenic.

The pulp may be destroyed after an injection of cocaine, but if the pulp is inflamed, the puncture of a syringe-point must be a decided objection to this operation. Besides, the action of cocaine preparations upon inflamed pulps is uncertain. My general practice is to treat a tooth for the reduction of the inflammation of the pulp, then by applying a sedative combined with the arsenic I generally succeed in the destruction of the pulp or a large part of it. In case the whole pulp is not destroyed by an application of arsenic, my plan is to rinse out the canal and make another application, unless the vital fibre remaining is very slight. If that is the case, a weak solution of chromic acid may be carefully applied, this will cause no sensation, providing the root is well closed at the apical foramen. Care has to be taken to prevent the chromic acid penetrating beyond the foramen. Then introduce a fine probe wound with a little cotton saturated with lime-water to neutralize the chromic acid. After that wipe out the root with eucalyptus oil or cajuput, or any non-irritating antiseptic, and stop it up for a test to see if it will remain free from inflammation before making a permanent filling. Sometimes I use what I call a rod-stopping for the root-canals. This I make of tin and lead. I have used wooden fibre and whalebone, but the wood and whalebone were soft, and a properly-shaped metallic rod answers the purpose very nicely. An antiseptic may be put in if you choose, and then the rod, dipped first in chloro-percha, is introduced, leaving length enough for easy manipulation. After inserting the rod-stopping, seal up the external cavity, and give the tooth a rest for several weeks. Give it plenty of time to heal before presuming to open it for the permanent filling.

If you need to give the tooth vent, you can pull out the rod-stopping without digging and working on a sore tooth. And yet, under ordinary conditions, the rod-stopping is just as good for security as the old-fashioned gold filling.

*Dr. Banfield.*—Do you use any particular proportion of bicarbonate of soda?

*Dr. Williams.*—No, sir; on a wisp of cotton take up as much as is convenient of apothecary's—not grocer's—bicarbonate of soda, and put it into the cavity, and cover it over with sandarach, to prevent the secretions from getting at it. It seems to reduce the inflammation more readily than anything I have ever tried. Where the pulp is resistant to the influence of arsenic, the bicarbonate-of-soda preparation seems, in some way, to help the destructive effect of arsenic. I have tried ammonia, but it doesn't appear to operate as well as bicarbonate of soda.

*Dr. Barker.*—There is one thought which has been frequently forced upon my mind in my practice, and that is, that both patient and operator are very apt to expect too much from the action of arsenic in an attempt to destroy the pulp. The previous speakers have noted the importance of reducing inflammation before we apply arsenic. The reduction of inflammation is ordinarily quite a simple matter, sedatives and antacids being useful in this connection. Very frequently we find the pulp inflamed and also strangulated. Simple depletion of the pulp in such a case may reduce the inflammation sufficiently to enable one to apply the escharotic with a reasonable promise of success. For a number of years I have noticed that most practitioners use arsenic in a fearful or timid way. They are afraid to allow arsenic to remain in contact with the pulp very long. Their rule being eight hours for ordinary cases, but, under no circumstances, longer than twenty-four. For a number of years, I have made it a rule not to remove the application under a week, frequently allowing it to remain in a month, and sometimes two or three months. The reason why dentists hesitate to allow arsenic to remain in contact with the pulp for more than twenty-four hours is because they are afraid of a sloughing or some complication they hardly know what. Where bad results have followed, it has been because the dentist has done his work carelessly, the arsenic being imperfectly sealed in the cavity. But if arsenic be applied carefully, after the adjustment of the rubber dam; if it be put in contact with the pulp and sealed in by something more than a mere dressing of cotton, saturated with sandarach varnish, there need be no fear of the result. For the last four or five years it has been my practice to apply arsenic to an inflamed pulp carefully, accurately, making the conditions as favorable for its destruction as I knew how, and then sealing it with a temporary stopping, and allowing the arsenic to remain there ten days. In the vast majority of cases, after such treatment, the pulp will be dead in all its parts.

Mention has been made of the use of tannic acid after arsenic has remained in contact with the pulp for a length of time. I have used tannic acid in my practice to harden and toughen the pulp, so that instead of being obliged to take it out piecemeal, you can drag it out entire.

Nearly all patients, when you tell them that you are going to kill a pulp, expect that they will experience no sensation whatever. They are usually disappointed.

The point I wish to emphasize in these remarks is that the majority of practitioners do not thoroughly seal up the arsenical application, and do not allow it to remain long enough in contact with the pulp.

*Dr. Williams.*—I would ask Dr. Barker if he would allow arsenic to remain as long in the tooth of a child as in that of an adult?

*Dr. Barker.*—Certainly not. Good judgment would require that you should not allow it to remain as long in a tooth whose foramen was very large, or in a deciduous tooth, as in an adult tooth.

*Dr. Williams.*—All we want to get rid of is the vitality in the pulp-chamber. The more vitality we can allow to remain in the tooth aside from that the better.

*Dr. Barker.*—Of course no one contemplates anything more than the destruction of the pulp. We do not seek to destroy the contents of the tubuli,—that would simply amount to the death of the entire tooth.

*Dr. Banfield.*—I would like to ask Dr. Barker if, after allowing his preparation to remain in three or four weeks, he finds a little soreness of the tooth on filling it?

*Dr. Barker.*—Whether I allowed it to remain twenty-four hours or three months, I should expect to find a slight tenderness on percussion.

Suppose we apply arsenic to the pulp of a superior incisor. The moment that pulp is dead, absolutely dead, to the apex, it imparts inflammation to the tissues just outside of the foramen, and you get a little peridental inflammation. The ensuing tenderness is almost pathognomonic of death of the pulp.

*Dr. Banfield.*—Whenever I have applied arsenic for the destruction of a pulp and the patient reports trouble, I at once nullify the influence of the arsenic, and make an application not only to remove the inflammation, but to make sure that it goes no farther.

*Dr. Williams.*—If inflammation of the tooth occurs after the application of arsenic, I at once employ some means for reducing it.

*Dr. Barker.*—A slight amount of peridental inflammation in such cases doesn't alarm me. I look upon it as an invariable accompaniment of the death of the pulp.

*Dr. Meriam.*—We ought to take cognizance of the inflammation that occurs in the presence of pulp stones, or the deposit of secondary dentine, which is extremely hard to penetrate, and where the use of anæsthetics would hardly avail us. As our remarks are read by men younger than ourselves, we should frankly state our difficulties.

We should also take cognizance of the extreme difficulty of removing many pulps quickly, and that pulps are not all formed on regulation lines; that the majority of those that require extirpation to-day are diseased or partially dead, and are in the molar teeth, and that there can be no royal road for their removal in the anterior roots of the first lower molars.

I have had several cases where, after arsenic had remained in a week, I have found sensitiveness, and in one case I worked up into the pulp-chamber and removed the secondary dentine at the entrance to the roots, taking out little plugs like stoppers of glass bottles.

I have found peroxide of hydrogen of advantage in the treatment of partially dead and inflamed pulps previous to the application of arsenic. I do not know that it has the permanent advantage of bicarbonate of soda, but it has a use previous to the application of soda.

*President Seabury.*—When the pulp is devitalized there must be a certain amount of inflammation in order to slough and cut off connections with surrounding parts.

*Dr. Smith.*—I regret that Dr. Briggs is not here to-night to elucidate his method of treating pulps, inflamed and otherwise, which require extirpation. You are probably familiar with a paper he read before the Harvard Odontological Society and published in the INTERNATIONAL DENTAL JOURNAL a few months ago. His method was to approach the pulp carefully, obtunding it by the use of a solution of cocaine, then by using a hypodermic syringe, on which he has substituted a blunt nozzle for the hypodermic point, to inject the cocaine into the pulp-chamber,—not into the pulp, but around the pulp. According to his report, he has in all cases been able to remove the pulp, fill the canal, and seal the cavity at the same sitting. Dr. Hamilton has also spoken of several successful operations in which he used this method. I have tried it in two cases, both of which were successful.

I have recently used Dr. Harlan's method of making an application of arsenic, iodoform, and cocaine mixed,—the proportions I cannot give you now from memory. This he allows to remain in the cavity for forty-eight hours, then he corrects the influence of the arsenic by the use of dialyzed iron, and then treats the pulp with an alcoholic solution of tannin, which he allows to remain for eight days. At the end of eight days your pulp is tanned, and is easily removed, and, if no secretions have gotten into the cavity, you can seal the canals with success. I have tried this method in several cases, and was successful.

*President Seabury.*—The next subject is, "To what Extent can Decalcified Dentine be left in Cavities of Decay when using Germicides?"

*Dr. Fillebrown.*—There should not be, in my judgment, much difference in the amount of tissue left, whether there be germicides used or not. The truth of that statement can be ascertained by considering for a moment the progress of decay, which is, perhaps, better described by Magitôt and Heitzmann than by any other authors. As I understand it, decalcification goes on to some considerable extent, and then the animal matter of the tooth is exposed to the action of the putrefactive bacteria which break down and destroy the matrix-substance. The discoloration extends into the dentine much farther than actual decay; and the microbes can, and do, force themselves into the tubuli of the teeth to a considerable extent beyond any softening, I should say to the depth of several lines. This is much farther than it is possible for any fluids to penetrate the tubuli of the teeth.

The point I wish to make is, that it is impossible to place in a cavity any substance, either in the form of a solution or otherwise, which will go far enough to reach all the destructive microbes. By dehydrating the tooth-substance, if that were possible, fluids could be made to penetrate more of the dentine than under normal circumstances. But still sufficient penetration could not be produced to reach all bacteria.

Therefore, you must depend upon something besides germicides to stop their action.

Dentine, when so far disorganized that it has lost its vital connection with the normal dentine of the tooth, cannot with safety be left in the cavity. It is claimed by some that it will regain its vitality after the decay has been arrested.

We know that decalcification may go on to a large extent and still the matrix may preserve its vitality, but dentine when softened and beginning to break down cannot be restored, and becomes an irritant instead of a protection, and is unsafe to leave in the cavity. Gutta-percha, cement, or some of the softer fillings, would be equally protective and less harmful to the pulp than disorganized dentine.

*Dr. Smith.*—I was led to present this subject for discussion from observing among my fellow-practitioners, especially among the young members of the profession, a great tendency to leave decalcified dentine, a punky condition of decay, in the cavity, and then fill it with oxychloride and oxyphosphate of zinc. The fact that they use the rubber dam and a strong germicide excuses them from thoroughly excavating the cavity, and they seem to feel that in this practice they are following the most advanced methods.

I was brought up in a different school, and was taught by that vigorous operator, Professor Moffatt, to thoroughly excavate.

Hard and discolored dentine might be left rather than expose a pulp, but not the leathery and punky dentine. The many cases of incomplete excavation of cavities which have fallen into my hands, teeth filled according to the method outlined above, do not bear out the statement that it is always a successful method. Operators who follow it do not excavate more because they believe that decay can go no farther after the use of germicides, and that the application of the germicide renders extraction unnecessary. If it does, then I am causing my patients unnecessary pain and taking up their time and my own. The remarks of Professor Fillebrown bear out my ideas on this matter: it is dangerous to leave this punky and leathery dentine in the cavity with the expectation that a germicide will prevent the further progress of decay.

*Dr. Williams.*—Cavities should be carefully excavated, and no morbid dentine should be left except such as is necessary to protect the pulp. If there has been no inflammation of the tooth, and you find a layer of softened, decalcified dentine over the pulp, there is no excuse for excavating that. To-day I gave a preliminary treatment to a tooth in which I simply excavated the borders, but the depth of the cavity was so very near to the pulp that I could jar it. The cavity was treated with mild antacids and antiseptics, and stopped up temporarily. This treatment will be renewed at short intervals, giving the pulp time to protect itself by a new deposit, so that by and by it can be permanently filled. I had a case, years ago, where I used this method, and which was alluded to in a paper before this society. It was the pulp of a molar which I thought was exposed. I could almost see the pulp, and a very slight pressure caused pain. It was in the case of a healthy young person of perhaps twenty-five, and was treated in a way that I described in that paper,—repeating mild applications at short intervals, the intervals growing longer as the tooth became harder, and allowing ten months to elapse after the last application. On opening the cavity it seemed the attempt was a failure; I thought I could see the pulp as at first, but on passing a probe down carefully, it was found there was a new dentine, transparent as a piece of glass, completely protecting the pulp. I remember lining that cavity with non-conducting material and filling it permanently. In the course of about three years afterwards the same tooth decayed on the distal side, and was very sensitive, showing that the pulp was still alive. If I had excavated rashly in this case there is not much doubt that there would have been a dead pulp. I think it is better to swab out the cavity with weak creosote and some slight antacid than to excavate almost to the surface of the pulp and put in osteoplastic with its strong acid, which hardens to a solid body and is relentless against the fine pulsations of the pulp.

*Dr. Meriam.*—We should take into consideration the time we wish a tooth to last. This would make a great difference in our treatment of the temporary and permanent teeth. The idea that an enduring operation can be built on an uncertain foundation should not occur to a well-trained dentist. If we spoke of germs as seeds I think we would understand this matter a little better. Now, what will destroy the life of a germ or seed? So far we have found but one real germicide, and I believe at Johns Hopkins some experiments have shown that for some germs even that (corrosive sublimate) is not a germicide.

If we would realize this and learn the time and treatment that a scientific man would devote to sterilizing any substance, and regulate our treatment by the knowledge thus gained, and expect to be governed by the principles involved, we should in time have some laws of practice to follow and not a mere "*ipse dixit.*" Dentists cannot work contrary to physical laws any more than can others. "Two bodies cannot occupy the same space at the same time." We cannot saturate with a germicide a substance that is already saturated with moisture, unless it can unite with it, and in uniting the germicide may be weakened below its working strength.

We should dehydrate if possible before using a germicide, to secure its penetration.

Nature seems to protect germs of all forms for reproduction. It is recorded that a bulb of Bulbocodium monophyllus showed life after being in a case of specimens, many of which were preserved with arsenic, for over twenty years. Some years ago I called the profession's attention to the coating of the teeth by mucus, and that it would in some cases prevent medicines acting on them, and I have recently been much interested in the experiment conducted at the Dead Sea with its water and the slime or ooze brought down by the stream that runs into it.

The water of the sea was tested in various parts and found to be absolutely sterile, but the mud drawn from its bottom contained germs that were isolated and cultivated. They were in water in which no animal or vegetable life exists, but being protected by the slime or ooze they have been found to retain their life. Now, in view of such retention of vitality, we should not attribute so much virtue to merely slushing a cavity with agents that are not germicides.

Some thirteen years ago, in treating the sensitive teeth of a child, I cauterized them with nitrate of silver, and afterwards without excavating filled with gutta-percha. This completely arrested decay. One of the teeth was afterwards extracted for regulating, and I had a good opportunity to study it. I hope that some time we shall encourage young men to devote some of their chemical training to our specialty, and some future Harvard man may give us a white caustic that will be sightly, and yet as effectual as the unsightly nitrate of silver is.

*Dr. Williams.*—Dr. Meriam's remarks about nitrate of silver remind me of a method which Dr. Keep tried many years ago when I was a student in his office.

He used nitrate of silver, and afterwards nitric acid, for cauterizing softened dentine which he wished to leave as a protection to the pulp. That process sterilized pretty effectually, but it also sometimes destroyed the vitality of the pulp.

The dehydrating of the tooth-substance, as Dr. Meriam says, is very essential in order to force medicinal applications into the tubuli. Applications can be made more penetrating by the addition of a little alcohol.

As to what Dr. Fillebrown said about germicides not penetrating the substance of the tooth, I think if he were to try first a solution of iron, and then, without removing the iron, apply tannin and insert a filling, he would find he had made ink in the tubuli, and I do not see why it is not reasonable to expect that our corrective agents may penetrate in the same way.

PRESENTATION OF SPECIMENS.

President Seabury said he had a syringe which he valued very highly. The barrel was made of glass, with a platinum point drawn out very fine, making it very serviceable for injecting fluids into root-canals. It also had the advantage that when it became clogged the point could be put in the flame of a lamp and the matter burned out without damaging the point.

Dr. Smith exhibited a set of taps and dies which were made, according to his directions, by S. W. Card, Mansfield, Mass. They were used in making screws, nuts, and jacks, especially in connection with regulating appliances, and he said the advantage which they had over those to be found at the dental depot was that they cut the right size of wire and also a much stronger thread. The set he showed included four different sizes and cut sixty-four threads to an inch, and one of them cut a left-handed thread, which he used in connection with the Shaw plate for spreading the lower arch.

Dr. Adams showed the model of a case of a nervous old gentleman where he had banded three loose teeth together and then made a plate to supply others that were lost. Two years afterwards their removal became necessary, and the band, together with the incrustation of tartar, made it possible to extract them all at once.

He also showed an appliance for holding the rubber dam in place. It consisted of a piece of piano-wire, three and a half inches long, bent to about a quarter circle, with a small knob at each end, and a loop in the middle. It is applied after the dam is in place, the latter being stretched over the ends and over the middle loop, which draws it away from the mouth in three directions, producing very much the same effect as the depressed dam.

Dr. Meriam read from the "Horticultural Year Book," published in England, an article on the preservation of fruits and vegetables by the insertion of plaster of Paris in decayed places, and Dr. Williams said that he remembered when a boy of seeing a farmer filling up decayed places in a lot of apples with slacked lime for the purpose of preventing further decay.

Dr. Meriam also presented a syringe which he had improvised by using an ordinary hard-rubber syringe and inserting a long piece of platinum and iridium tubing in place of the usual point. It had served admirably in treating a fistulous tract.

William H. Potter, D.M.D.,
*Editor American Academy of Dental Science.*

